# Arterial oxygenation and acid–base status before and during oxygen supplementation in captive European bison (*Bison bonasus*) immobilized with etorphine-acepromazine-xylazine

**DOI:** 10.3389/fvets.2023.1125919

**Published:** 2023-06-12

**Authors:** Nino Gardoni, Sven Björck, Jacopo Morelli, Alina L. Evans, Daniela S. B. Barros, Rikard Wiklund, Anne Randi Græsli, Alexandra Thiel, Jon M. Arnemo, Marianne Lian

**Affiliations:** ^1^Department of Forestry and Wildlife Management, Faculty of Applied Ecology, Agricultural Sciences and Biotechnology, Inland Norway University of Applied Sciences, Campus Evenstad, Koppang, Norway; ^2^Avesta Visentpark, Avesta, Sweden; ^3^Skeldale Veterinary Hospital—Medivet Thirsk 24h, Thirsk, United Kingdom; ^4^Department of Wildlife, Fish and Environmental Studies, Faculty of Forest Sciences, Swedish University of Agricultural Sciences, Umeå, Sweden

**Keywords:** arterial blood gas, chemical immobilization, etorphine hydrochloride, hypoxemia, respiratory acidosis, ungulate, wisent, xylazine hydrochloride

## Abstract

Chemical immobilization of captive European bison (*Bison bonasus*) is often required for veterinary care, transportation, or husbandry practices playing an important role in conservation breeding and reintroduction of the species. We evaluated the efficiency and physiological effects of an etorphine-acepromazine-xylazine combination with supplemental oxygen in 39 captive European bison. Animals were darted with a combination of 1.4 mg of etorphine, 4.5 mg of acepromazine, and 20 mg of xylazine per 100 kg based on estimated body mass. Arterial blood was sampled on average 20 min after recumbency and again 19 min later and analyzed immediately with a portable i-STAT analyzer. Simultaneously, heart rate, respiratory rate, and rectal temperature were recorded. Intranasal oxygen was started after the first sampling at a flow rate of 10 mL.kg^−1^.min^−1^ of estimated body mass until the end of the procedure. The initial mean partial pressure of oxygen (P_a_O_2_) was 49.7 mmHg with 32 out of 35 sampled bison presenting with hypoxemia. We observed decreased respiratory rates and pH and mild hypercapnia consistent with a mild respiratory acidosis. After oxygen supplementation hypoxemia was resolved in 21 out of 32 bison, but respiratory acidosis was accentuated. Bison immobilized with a lower initial drug dose required supplementary injections during the procedure. We observed that lower mean rectal temperatures during the immobilization event were significantly associated with longer recovery times. For three bison, minor regurgitation was documented. No mortality or morbidity related to the immobilizations were reported for at least 2 months following the procedure. Based on our findings, we recommend a dose of 0.015 mg.kg^−1^ etorphine, 0.049 mg.kg^−1^ acepromazine, and 0.22 mg.kg^−1^ xylazine. This dose reduced the need for supplemental injections to obtain a sufficient level of immobilization for routine management and husbandry procedures in captive European bison. Nevertheless, this drug combination is associated with development of marked hypoxemia, mild respiratory acidosis, and a small risk of regurgitation. Oxygen supplementation is strongly recommended when using this protocol.

## Introduction

Captive breeding of European bison (*Bison bonasus*), also called wisent, played an important role in saving the species from total extinction and still help maintaining genetic variation from a small genetic base. These breeding programs often require chemical immobilization and manipulation for veterinary care, transportation or other husbandry practices (1–3). However, the size and strength of European bison make physical restraint very dangerous for both the animal and the handler (2, 4). Although, hand injections in a hydraulic chute have been reported for American bison (*Bison bison*) (5–8), the use of dart gun and chemical immobilization is considered safer (2, 9). Using a remote drug delivery system allows intramuscular injection with minimal physical manipulation of the animal, reducing the animal’s stress and physical exertion, compared to physical restraint, as well as reduced risk for veterinarians and handlers (10). Yet, chemical immobilization still involves a risk of physical trauma to the animal related to darting (11) and requires professionally trained and experienced personnel (12). Prior examination and weighing of the animal cannot generally be done beforehand, increasing the risk of overdosing, meaning the administration of more drug than desired, or underdosing, meaning the administration of less drug than required, as well as other complications inherent to chemical immobilization (13, 14). The main complications reported in American bison during chemical immobilization are hypoxemia, bloat, and regurgitation (6, 15, 16). As with many other ruminant species, bison stress easily (15) which can predispose them to capture myopathy, a rhabdomyolysis induced by stress and muscular exertion that affects skeletal and cardiac muscles and can lead to death in some case (17, 18). As such, there is a need to document the efficacy and outcomes of chemical immobilization procedures on European bison to prevent unexpected events and reduce negative impacts.

Several protocols have been used for bison immobilization. The combination of an alpha-2 adrenoceptor agonist with either tiletamine-zolazepam (1, 5, 6, 19) or ketamine (5, 19–22) was reported as efficient for both American bison and European bison. Azaperone and medetomidine in combination with either nalbuphine (8) or butorphanol (16) were also evaluated as good alternatives for American bison immobilization. Additionally, standing sedation using butorphanol and detomidine was reported for European bison (23). Finally, the combination of a potent opioid and an alpha-2 adrenoceptor agonist is efficient for immobilization of a wide range of ungulate species (20, 24). Currently, the use of etorphine with either acepromazine (3, 25, 26), xylazine (3, 19, 20), or both (3) is the most common drug combination reported for European bison immobilization (9).

Etorphine is an ultra-potent opioid agonist providing analgesia and dose-dependent sedation (27) allowing for a rapid immobilization with a relatively high margin of safety and with several efficient total or partial competitive antagonists available (28). Acepromazine, a phenothiazine, is combined with etorphine for its anxiolytic effects in Large Animal Immobilon® (29). Xylazine is an alpha-2 adrenoceptor agonist producing a dose-dependent sedation, analgesia and muscle relaxation (30, 31). The combination of these drugs potentiates their effect allowing reduced volume and mitigating some side effects of etorphine such as excitement or muscle rigidity (28, 29). Xylazine is, additionally, fully reversible (30). Nevertheless, several risks remain associated with etorphine-acepromazine-xylazine immobilization of wild ungulates, including bloat, ruminal tympany, regurgitation (24), hyperthermia (28, 32), re-narcotization (13, 28), and cardiovascular effects (mostly bradycardia and hypotension due to xylazine) (30, 33). Hypoxemia, and consequent hypoxia, associated with hypoventilation, and secondary acid–base disturbances are also common complications of ruminant immobilization using this combination (13). Both etorphine (28, 31) and xylazine (30, 32, 34) affect blood oxygenation and their combination, with or without acepromazine, has been reported to induce hypoxemia and metabolic disorders in moose (*Alces alces*) (35, 36), muskox (*Ovibos moschatus*) (37), reindeer (*Rangifer tarandus*) (38), rhebok (*Pelea capreolus*) (39), and scimitar horned oryx (*Oryx dammah*) (40). Prolonged hypoxemia and metabolic disorders can lead to some level of organ dysfunction, such as postanesthetic renal or cardiac insufficiency (41), and can affect recovery (13, 38). In combination with hyperthermia and an increased tissue oxygen consumption following induction, hypoxemia can also increase the risk of inducing capture myopathy (18). To prevent such events, it is recommended to monitor arterial blood gases and acid–base status (42). Improvement of oxygenation using supplemental oxygen has been successfully implemented in several ruminant species (36–38, 43, 44) including American bison (8).

To our knowledge, very few studies on bison have focused on the physiological responses to chemical immobilization (6, 8) and none have documented blood oxygenation and acid–base status of European bison immobilized with etorphine, acepromazine, and xylazine. Based on this we conducted a physiological evaluation of etorphine-acepromazine-xylazine immobilizations in European bison. The aims of our study were to: (1) document the efficiency of captive European bison immobilization using a combination of etorphine-acepromazine-xylazine. Specifically, we investigated efficient doses providing sufficient level of immobilization for routine procedures without supplemental injections, and we reported induction, handling and recovery times. (2) report clinical and physiological effects for heart rate, respiratory rate, rectal temperature, and arterial blood gas. (3) evaluate benefits of oxygen supplementation during the immobilization, by comparing blood gas and clinical parameters before and during oxygen supplementation. (4) Assess if hypoxemia affects recovery time.

## Materials and methods

### Ethics approval

All samples used in this study were collected as part of a standardized routine animal anesthesia monitoring protocol during chemical immobilization of bison, either for management, veterinary care or for translocation purposes. Because the samples were collected as part of standardized monitoring protocols during immobilizations, needed by compulsory EU health regulations and approved by Avesta Visentpark, the study did not require further ethical approval.

### Animals and study area

We conducted the study during six different sessions in 2013, 2014, 2017, 2019, 2021, and 2022, each event lasting 1–5 days, between the 18 April and 1 June, at Avesta Visentpark (AVP) in Sweden. AVP is one of the oldest breeding centers in Europe strictly dedicated to conservation breeding of European bison of the Lowland Caucasian line. It hosts two breeding herds including 20–40 individuals in total. All the bison are born in captivity and bred in large enclosures for reintroduction purpose, to increase wild population and maintaining rare genetic founder line for future captive and wild populations. During the immobilizations, the atmospheric pressure (mean ± SD) was 754 (±7) mmHg, and the ambient temperature was 11.6 (± 5.8)°C. The study included 39 captive bison, 25 females (64%) and 14 males (36%), 1–10 years old, and weighing 125–800 kg (362 ± 144 kg, *n* = 28). Immobilizations were done either as part of management procedures required for bison translocation to Romania for reintroduction purposes (2), or for routine husbandry procedures.

### Induction

Two days before the immobilizations we split all animals into smaller enclosures containing two to three bison. We fasted all of them either the morning or the evening before the immobilization event to reduce the risk of regurgitation and tympany (45). We then isolated animals in individual enclosures before immobilizing them, one by one, with a combination of 1.38 mg of etorphine base and 4.5 mg of acepromazine base per 100 kg (Large Animal Immobilon®, Novartis Animal Health, Frimley, United Kingdom; 2.25 mg.mL^−1^ etorphine, 7.38 mg.mL^−1^ acepromazine) and 20 mg xylazine base for 100 kg (Rompun®, Bayer AG, Leverkusen, Germany, 500 mg). For most animals, we previously estimated the body mass and confirmed it with weighing during the immobilization procedure using an electronic scale, either on the same day or 3 days later during loading, for animals being translocated.

The drug combination was injected intramuscularly (IM) in the hindquarter, using an 11 mm CO2 powered rifle and a 1.5 or 3 mL dart syringe with a 2 mm × 40 mm needle with a shorten barb and double-ported (DANiNJECT ApS, Kolding, Denmark). We monitored clinical signs of chemical immobilization and if recumbency was not achieved by 10–20 min, we administered a second dose by darting or hand injection. We adjusted supplementary doses, from 25 to 100% of the first dose, based on the apparent depth of immobilization.

We recorded the time from darting to the first sign of effect of chemical immobilization and induction time (time from darting to recumbency). Once the individual was down, we positioned it in sternal recumbency with head held up and nose pointing down, when possible, and applied a blindfold. In some cases, animals were pulled on a sled by an all-terrain vehicle to a more suitable enclosure for the procedure and the recovery, leading to large variation between individuals in timing before sampling could be done and oxygen supplementation initiated.

### Arterial blood sampling and analysis

As soon as possible after sternal recumbency and again 15 (±4) min after the start of oxygen (time since recumbency recorded as t1 and t2), we sampled between 0.5 and 1 mL arterial blood anaerobically from the auricular artery. For sampling, we used a 1 mL pre-heparinized syringe (Portex®, Smith medical ASD Inc., Keene, United States) and a 23-gauge needle. We immediately mixed the whole blood with the anti-coagulant by rolling the syringe in the palm and discarded the two first blood drops before it was analyzed using an i-STAT®1 Portable Clinical Analyzer and i-STAT® CG4+ cartridges (Abbott Laboratories, Illinois, United States). We maintained the analyzer and the cartridges at operating temperature (16–30°C) in an insulated box filled with warmed water bottles, as needed based on ambient temperatures.

For the arterial blood gas and acid–base analysis, the partial pressure of oxygen in arterial blood (P_a_O_2_), the partial pressure of carbon dioxide (P_a_CO_2_), pH and lactate were measured while the base excess (BE) and bicarbonate (HCO_3_^−^) were calculated. We measured rectal temperature at the sampling time and used it for correction of the blood gas and pH values for both samples (46). Based on temperature corrected values, we defined hypoxemia as mild (P_a_O_2_ = 60–80 mmHg), marked (P_a_O_2_ = 40–60 mmHg), or severe (P_a_O_2_ < 40 mmHg). We defined hypercapnia as mild (P_a_CO_2_ = 50–60 mmHg), marked (P_a_CO_2_ = 60–80 mmHg), and severe (P_a_CO_2_ > 80 mmHg). We defined acidemia as pH < 7.35 and considered it as marked for pH < 7.2.

### Monitoring and animal handling

After the first arterial blood sample was collected, we positioned a soft flexible plastic cannula in one nostril and advanced the tip to the level of the medial canthus of the eye and secured it with medical tape (47). A flow of 100% oxygen concentration was delivered by an oxygen cylinder through this cannula at a flow rate of approximately 1 L.min^−1^ for every 100 kg based on the estimated body mass. We recorded the time when the oxygen was started.

We monitored several physiological parameters throughout the immobilization and recorded these just after the animal was approached and again 10–15 min later. We monitored heart rate either by direct auscultation with a stethoscope or indirectly by palpation of the auricular artery. We assessed the capillary refill time and the mucous membrane color from the gingiva, the respiratory rate by counting thoracic elevation, and we measured the rectal temperature with a digital thermometer. Hyperthermia was define for rectal temperature recorded 2°C above normal rectal temperature (20), considered close to 38.5°C in European bison (48, 49). For bison presenting with regurgitation, 30 mg.kg^−1^ of body mass of a long acting oxytetracycline formulation was administered, given 2/3 IM and 1/3 SC (Tetroxy prolongatum vet® 200 mg.mL^−1^, Ceva Animal Health, Lund, Sweden).

During the immobilization, a long-acting neuroleptic was administered to the animals that were scheduled for translocation 3 days later, as a part of a re-wilding program. We used 200 mg per 100 kg of body mass of Cisordinol-Depot® IM (Lundbeck Pharma A/S, Valby, Denmark, zuclopenthixol decanoate 200 mg.mL^−1^). The onset of action of this neuroleptic is several days so we did not consider it to interfere with our study (22, 50).

### Reversal

After all management and husbandry procedures, we discontinued oxygen supplementation and immediately antagonized the immobilizing agents with 1.35 mg diprenorphine IM (Large Animal Revivon®, Novartis Animal Health, Frimley, United Kingdom, 3 mg.mL^−1^ diprenorphine) per 1 mg etorphine and 1 mg atipamezole IM (Antisedan®, Orion Pharma Animal Health, Turku, Finland, 5 mg.mL^−1^ atipamezole) for every 8 mg xylazine. We recorded the time when O_2_ delivery was stopped, the time of antagonist administration, the first sign of recovery, and the time the animal was standing. We defined the recovery time as the time between the antagonist administration and the time the animal was fully standing. Animals were observed regularly during the 24 h following immobilization for any adverse effects.

### Statistical analysis

#### Sample size

Due to the opportunistic aspect of the study, we conducted the analyses on different sample sizes. We excluded samples from the analysis due to sample contamination, absence of a paired sample, missing body mass, discontinuity during oxygen delivery, early reversal for animal safety reasons, or use of an alternative reversal agent (naltrexone).

#### Need for supplemental injections

We calculated the summary statistics [reported as mean ± SD (min – max)] for the total doses of etorphine, acepromazine, and xylazine administered throughout the whole procedure.

To study the relationship between the need for supplemental injection and the initial dose injected with the first dart, we split the bison into two groups depending on whether they had received only one injection or several injections. We modeled the dose injected with the first dart as a function of the group with a simple linear model. The full results of the regression analysis are reported in Additional File 1.

#### Descriptive analyses of the physiological variables

We calculated the summary statistics for each physiological variable [i.e., rectal temperature, pulse rate, respiratory rate, P_a_O_2_, P_a_CO_2_, pH, HCO_3_^−^, base excess (BE), and lactate] for both samples (t1 and t2).

#### Change in physiological parameters between t1 and t2

The normality of the distribution for each parameter was assessed both graphically and by using a Shapiro–Wilk test on the values from both samples and from the difference between the two measurements. For normally distributed data, we used a two-tailed paired *t*-test to analyze the variation between the first and the second sample. For those that violated the normality assumption, we used the non-parametric Wilcoxon signed-rank test for paired data. For every comparison, we reported the mean difference ( $\bar{x}$ _t2-t1_), the T-or V-statistic of the test with the degree of freedom (T_df_ and V_df_) and the value of *p* (*p*).

#### Immobilization time

We calculated the summary statistics for the duration of different phases of the immobilizations. To assess factors that potentially affected the recovery time, we used generalized linear models with a gamma distribution and log-link function. To reduce the risk of overfitting, regarding our sample size and because of the presence of collinear covariates, we first modeled the recovery time as a function of either the mean P_a_O_2_ of the procedure, the mean rectal temperature, the procedure time (defined as the time in minutes from the moment the animal was recumbent to the time the antagonist was administered), the total dose of etorphine received, or the total dose of xylazine received (both in mg.kg^−1^). Then, we applied a forward selection method based on the small sample corrected Akaike Information Criterion (AICc). In the case of ∆AICc <2 between two models, we chose the one with the lowest degree of freedom according to the principle of parsimony (Additional File 2). In the results, we reported the sample size, the regression coefficient (β), and the 95 percent confidence interval (95% CI) for the best model and after the back-transformation of its coefficients using the exponential function to help its interpretation. The full results of the regression analysis are presented in Additional File 1.

All the statistics were done using R, version 4.1.2 (51). For all the analyses, the level of significance was set to 0.05. For each model, we verified the assumptions by using the DHARMa residuals diagnostic tools from the package DHARMa (52).

## Results

**Figure 1:**
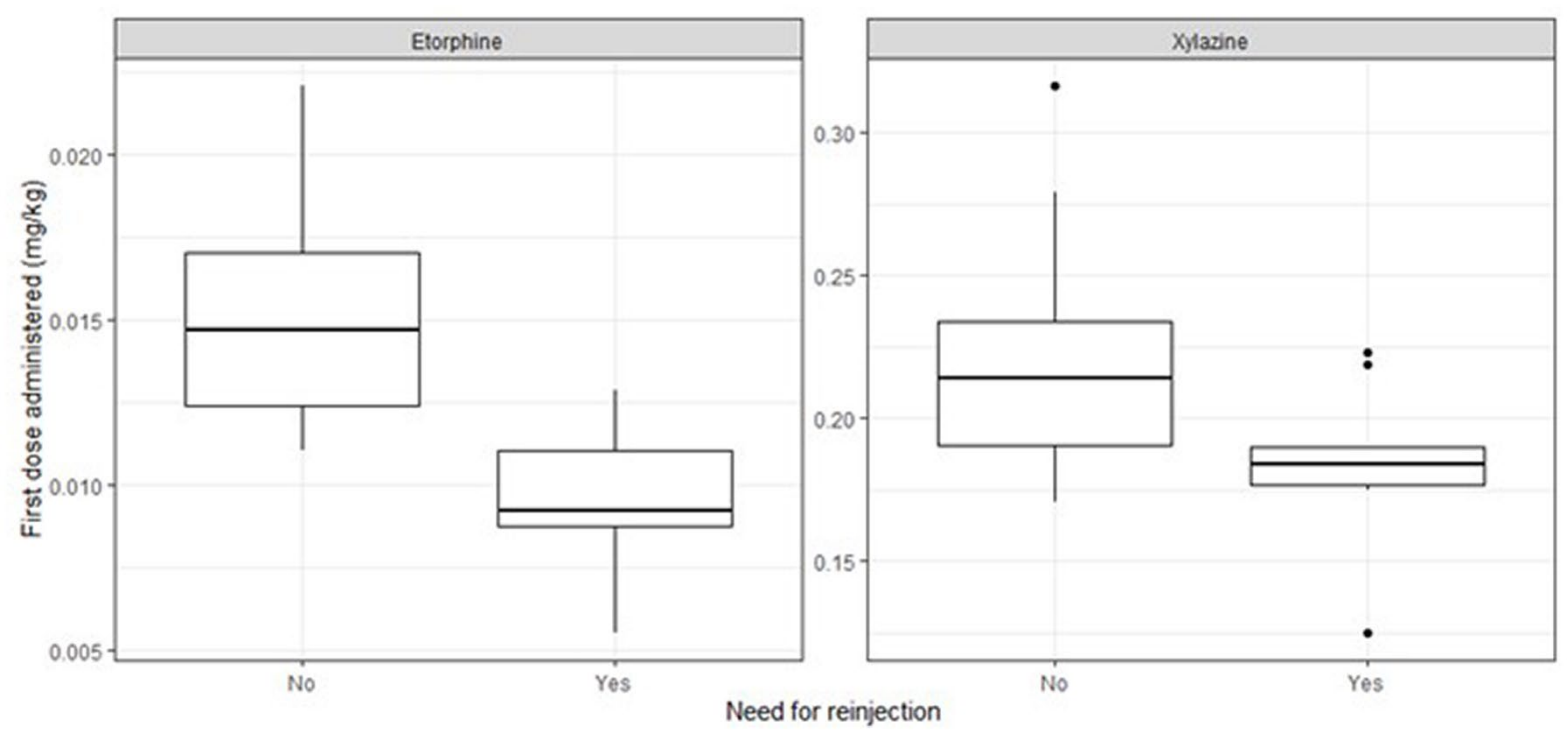
The initial dose of etorphine and xylazine administered for individuals that needed supplemental injections (Yes) or not (No).

During six sessions from 2013 to 2022, we immobilized 39 bison as part of scheduled health maintenance, and pre-translocation procedures. The average total dose used was 0.015 ± 0.003 (0.010–0.022) mg.kg^−1^ of etorphine and 0.048 ± 0.009 (0.033–0.072) mg.kg^−1^ of acepromazine combined with 0.25 ± 0.09 (0.17–0.48) mg.kg^−1^ of xylazine IM (*n* = 26). The first dart provided a sufficient level of immobilization for the whole procedure for 25 bison (64%), while 14 (36%) required 1–3 supplemental injections to complete the procedure. Among the latter, five (12.8%) received extra doses of etorphine-acepromazine, one (2.6%) received an extra dose of xylazine and eight (20.5%) received both. Bison that required extra doses received significantly smaller initial dose of etorphine (*n* = 26; β = −0.006; 95% CI [−0.008, −0.003]) and xylazine (*n* = 26; β = −0.04; 95% CI [−0.07, −0.01]) from the first dart, than the bison not requiring extra doses (Figure 1). The induction, procedure, and recovery times and the variations between individuals are reported in Table 1.

**Table 1:** Induction, procedure, and recovery times for captive European bison immobilized with etorphine-acepromazine-xylazine.

Time (min)	*n*	Mean	SD	min - max
Induction	39	6.6	4.7	1.0–22.0
Procedure	35	49.1	13.5	24.0–71.0
Recovery	34	11.4	7.5	2.0–38.0

The induction time corresponds to the time between darting and recumbency. The procedure time corresponds to the time from recumbency to reversal. The recovery time corresponds to the time from reversal to standing. All times are reported in minutes.

We successfully analyzed the initial blood gas sample for 35 bison, four individuals were not included due to sampling issues. We successfully conducted the second blood gas analysis on 32 individuals; seven animals were not included due to either problems with oxygen delivery or sampling issues. We compared the two samples for the 31 bison where both results were available. The physiological parameters and blood gases we measured before and after the start of O_2_ administration are reported in Table 2. Before the start of oxygen delivery, the mean P_a_O_2_ was 49.7 ± 18.3 (21–101) mmHg. Thirty-two bison (91%) presented with a P_a_O_2_ < 80 mmHg (21–79). Among these, nine (26%) had a P_a_O_2_ < 40 mmHg, 18 (51%), a P_a_O_2_ between 40 and 60 mmHg and five (14%), and a P_a_O_2_ between 60 and 80 mmHg. Hypercapnia was present in 24 bison (69%; 50–64 mmHg). The pH was between 7.30 and 7.35 for 15 bison (43%) and 7.22 for one (3%). The mean lactate was 1.18 ± 0.90 (0.30–4.02) mmol.L^−1^, and the mean bicarbonate concentration was 28.8 ± 3.1 (22.3–34.5) mmol.L^-1^.

**Table 2:** Physiological parameters from captive European bison (*Bison bonasus*) during chemical immobilization using etorphine-acepromazine-xylazine.

Variable	Unit	*n_t1_*	Mean ± SD (min – max)	Trend	*n_t2_*	Mean ± SD (min – max)	*p*-value *(n)*
Pulse rate	Beats.min^–1^	39	54 ± 8 (32–76)	=	29	52 ± 9 (32–72)	0.313^1^ (*n* = 28)
Respiratory rate	Breath.min^–1^	39	11 ± 8 (2–36)	=	29	9 ± 4 (4–16)	0.177^2^ (*n* = 28)
Rectal temperature	°C	39	38.7 ± 0.6 (37.3–39.7)	=	32	38.7 ± 0.6 (37.5–39.8)	0.442^2^ (*n* = 31)
pH ^*a^		35	7.36 ± 0.05 (7.22–7.48)	↘	32	7.33 ± 0.05 (7.22–7.46)	< 0.001^1^ (*n* = 31)
P_a_O_2_^*a^	mmHg	35	49.7 ± 18.3 (21.0–101.0)	↗	32	90.2 ± 28.3 (49–165)	< 0.001^1^ (*n* = 31)
P_a_CO_2_^*a^	mmHg	35	52.2 ± 7.5 (36.1–64.3)	↗	32	59.0 ± 8.2 (36.3–71.8)	< 0.001^1^ (*n* = 31)
HCO_3_^–a^	mmol.L^–1^	35	28.8 ± 3.1 (22.3–34.5)	↗	32	30.1 ± 3.4 (21.4–36.0)	0.003^2^ (*n* = 31)
BE	mmol.L^–1^	35	7.2 ± 10.8 (–3.0–36.0)	=	32	4.0 ± 3.4 (–5.0–10.0)	0.653^2^ (*n* = 31)
Lactate^a^	mmol.L^–1^	34	1.18 ± 0.90 (0.30–4.02)	↘	32	0.85 ± 0.54 (0.30–2.42)	0.001^2^ (*n* = 30)

^*^Temperature corrected values; ^a^significant difference after oxygen supplementation. t_1_ and t_2_ correspond to the time from when the animal is down to respectively the 1st and the 2nd sample. ∆_t_ is the time between the first and the second sample. For every parameter, the mean ± SD (min-max) and the number of animals (*n*_t_) are reported. The *p*-values are the results of paired *t*-tests between t_1_ and t_2_ for each parameter on n individuals. ^1^two-tail paired t-test; ^2^Wilcoxon signed-rank test.

P_a_O_2_, partial pressure of oxygen in arterial blood; P_a_CO_2_, partial pressure of carbon dioxide in arterial blood; HCO_3_^–^, concentration of bicarbonate in arterial blood; BE, Base excess.

**Figure 2:**
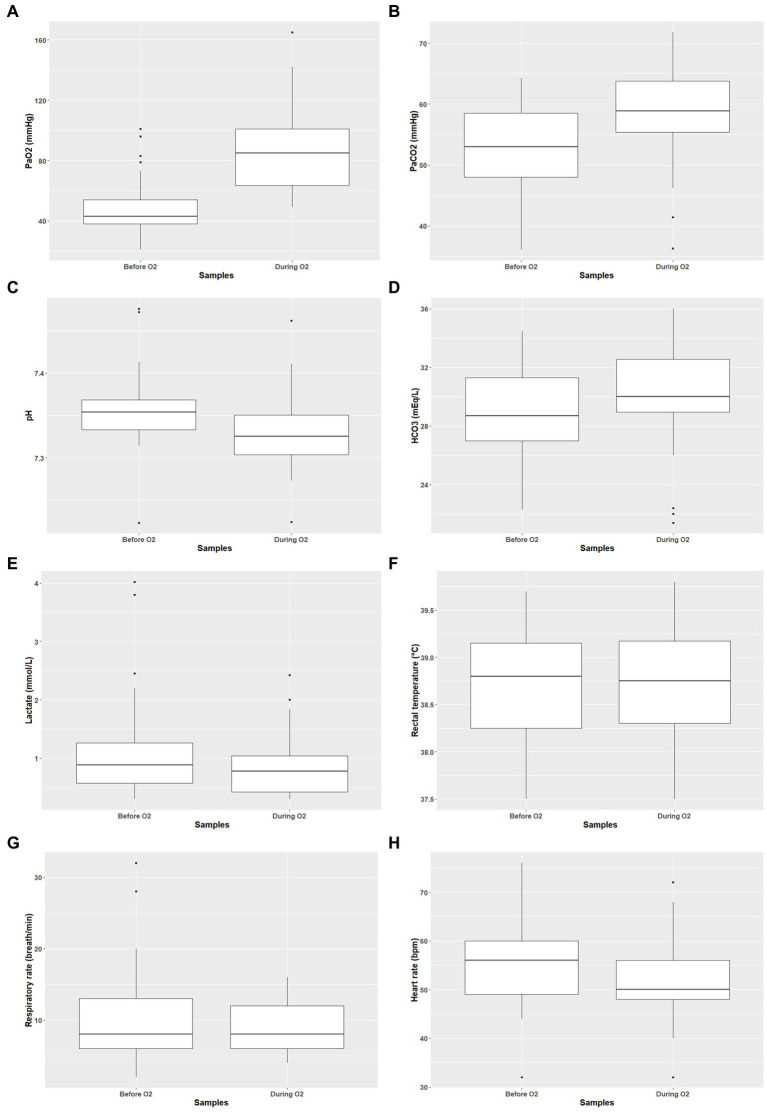
Arterial blood gases and clinical parameters before and during oxygen supplementation Presentation of P_a_O_2_ **(A)**, P_a_CO_2_ **(B)**, pH **(C)**, bicarbonate blood concentration **(D)**, lactate blood concentration **(E)**, rectal temperature **(F)**, respiratory rate **(G)**, and heart rate **(H)** before and during oxygen supplementation.

After 15.4 ± 3.7 (9.0–23.0) min (*n* = 30) with 12 ± 5 (7–32) mL.kg^−1^.min^−1^ of oxygen supplementation (*n* = 22), the P_a_O_2_ ( $\bar{x\;}$ _t2-t1_ = 39.8 mmHg; T_30_ = 7.0; *p* < 0.001), P_a_CO_2_ ( $\bar{x\;}$ _t2-t1_ = 5.9 mmHg; T_30_ = 4.2; *p* < 0.001), and bicarbonate levels ( $\bar{x\;}$ _t2-t1_ = 1.1 mmol.L^−1^; V_30_ = 379; *p* = 0.003) increased significantly, while pH ( $\bar{x\;}$ _t2-t1_ = −0.03; T_30_ = −4.0; *p* < 0.001) and lactate ( $\bar{x\;}$ _t2- t1_ = −0.30 mmol.L^−1^; V_29_ = 62.5; *p* = 0.001) decreased significantly (Figure 2). Of the 32 bison sampled after oxygen supplementation, 10 had hypoxemia (31%), 27 hypercapnia (84%), 23 acidemia (72%), and one hyperlactatemia (3%). No significant differences were found in pulse rate, respiratory rate, and temperature, before and after oxygen supplementation. We did not observe hyperthermia during the study.

**Figure 3:**
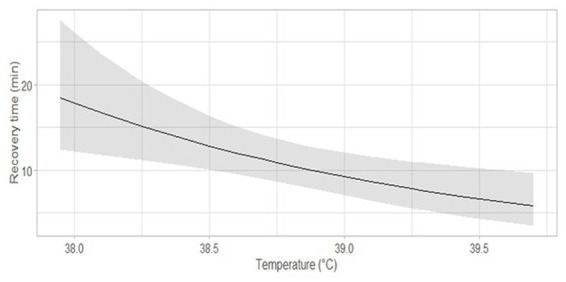
Predicted recovery time as a function of the mean rectal temperature through the immobilization procedure. The dark gray area around the curve corresponds to the 95% confidence interval.

The recovery time was negatively associated with the mean rectal temperature during the procedure (*n* = 33; β = 0.62; 95% CI [0.44, 0.86]; Figure 3). We did not identify a significant association between recovery time and mean P_a_O_2_, procedure time, etorphine, or xylazine dose. Three of the immobilized bison presented with small amount of ruminal fluid in the mouth and the nostrils during the procedure and two had rough recoveries characterized by either hyperexcitation or marked difficulty to recover balance. We did not observe bloating during the study. For the three bison presented with minor regurgitation, an injection of long-acting tetracycline was administered. No mortality was reported for at least 2 months post procedures.

## Discussion

Here we present a protocol for chemical immobilization of captive European bison using a combination of etorphine-acepromazine and xylazine. Our main results indicate faster induction times and recumbency when using one dart with a higher initial dose of etorphine-acepromazine and xylazine, severe hypoxemia which was alleviated after nasal oxygen insufflation, and slower recovery times for animals with decreasing body temperatures. Observation of decreased respiratory rate, decreased pH and mild hypercapnia characterized respiratory acidosis induced by chemical immobilization and possibly intensified by oxygen supplementation. By using this combination, we obtained an efficient and reversible immobilization with a controlled recovery for all bison in our study. This protocol provided a quick induction with a mean induction time (6.6 ± 4.7 min) faster than induction times reported for ground-darted American bison using combination of butorphanol-azaperone-medetomidine (10.8 ± 7.3 min) (16), nalbuphine-azaperone-medetomidine (11.5 ± 1.3 min) (8), or carfentanil-xylazine (14.2 ± 2.9 min) (54) and close to xylazine-zolazepam-tiletamine (4.1 ± 1.0 min) and medetomidine-zolazepam-tiletamine (7.5 ± 2.1 min) protocols (6). However, we recorded variations in induction time (1–22 min), reflecting a variable number of supplemental injections. Such lack of consistency that we discovered in captive individuals could translate into an increased unpredictability in induction time if this drug combination was used to immobilize free-ranging bison.

The average dose of etorphine required was similar to the dose previously reported for European bison (0.015 mg.kg^−1^), but we required a slightly higher dose of xylazine (0.25 mg.kg^- 1^ instead of 0.20 mg.kg^−1^) (20). Also, we observed that when lower doses were administered in the first dart, due to inaccuracies in estimating the body mass, we usually had to administer a supplementary dose either to reach or prolong a safe level of immobilization. Although lower dosages for this combination of drugs have been reported to provide safe immobilization on European bison (3), underdosed animals are a risk for handlers and themselves as bison can suddenly arouse (S. Björck, personal observation, 2022). Additionally, delayed induction time after underdosing could lead to overexcitation and stress and increase the risk of trauma (15, 28), capture myopathy, and likely lead to a higher drug requirement (13). Etorphine is considered as relatively safe for Bovidae species (55) and doses up to 0.03–0.05 mg.kg^−1^ on European bison have been reported without associated mortality or severe side effects (26). Xylazine presents a narrower safety margin, but reports show dosages exceeding 3–10 times our dosage in several ruminant species without mortality (56). Considering these relatively broad safety margins, together with the availability of specific antagonists allowing for quick reversal for both drugs in case of emergency (29), we recommend personnel working with European bison, when the weight is unknown, to administer high dose to the animal, rather than aiming for the minimal effective dosage risking underdosing and re-darting. Using our recommendation can possibly augment the variation we observed in induction times. In our study, the doses we used in the group that did not require supplementary injections were 0.015 mg.kg^−1^ etorphine, 0.049 mg.kg^−1^ acepromazine, and 0.22 mg.kg^−1^ xylazine.

Hypoxemia is generally considered one of the main concerns during American bison immobilization because of its severity and frequent occurrence (15). Mild to marked levels of hypoxemia was previously reported in American bison immobilized with different drug combinations [xylazine-tiletamine-zolazepam, P_a_O_2_ = 53 mmHg (6); medetomidine-tiletamine-zolazepam, P_a_O_2_ = 58 mmHg (6); butorphanol-azaperone-medetomidine, P_a_O_2_ = 62 mmHg (57); nalbuphine-medetomidine-azaperone, P_a_O_2_ = 64 mmHg (8)]. Several causes can lead to its development such as hypoventilation or drug-induced intrapulmonary problems (ventilation-perfusion mismatch, shunt, or diffusion impairment) (47). In large mammals, recumbency is known to contribute to hypoxemia through the pressure applied by the abdominal viscera on the diaphragm (41). In our study, all animals were positioned in sternal recumbency and, although it is considered as favoring better oxygenation than lateral and dorsal recumbency in several species (58–60), it probably contributed to the development of hypoxemia (41). Both etorphine and xylazine are respiratory depressants (33), and have been associated with hypoxemia during immobilization of ruminants (35–40). Etorphine is reported to decrease the responsiveness of central chemoreceptors to the variation in P_a_CO_2_ (29) inducing a dose-dependent respiratory depression in ruminants resulting in hypoventilation (28, 31). A similar effect of xylazine on central responsiveness to CO_2_ is reported (29). Previous studies on ungulates have suggested hypoventilation and potentially ventilation-perfusion mismatch as major contributors to hypoxemia during immobilization using etorphine and xylazine (35, 36, 58). In our study, we observed a lowered mean respiratory rate [physiological range: 10–20 breath.min^−1^ (48, 61)] and a mild hypercapnia [physiological range: 30–50 mmHg (22)], reflecting hypoventilation (41). Therefore, the development of hypoxemia in our study likely results from the observed hypoventilation, sternal recumbency, and potential ventilation-perfusion mismatch induced by the drugs administered.

After intra-nasal oxygen delivery, we observed a significant increase in mean P_a_O_2_ with values reaching the physiological normal range for 21 out of the 31 bison sampled twice. Non-invasive oxygen supplementation is recognized as a quick, easy and safe method to improve arterial oxygenation in animals that experience ventilation-perfusion mismatching and hypoventilation (47, 62), and former studies reported its successful implementation in several ungulate species (35–39, 43, 44). The presence of hypoxemic bison after oxygen treatment is most likely explained by a too low delivery flow of oxygen. Recommended flow rates, of 10–15 L.min^−1^, are reported for American bison (15). Yet there is a lack of data and study available to assess an efficient flow rate for bison. Based on personal experience with other large wild ungulates (35–37, 63, 64), we started with 10 mL.kg^−1^.min^−1^ (1 L.min^−1^ per 100 kg). Although, we titrated up the flow rates early in the study and ended up with an average flow rate of 12 mL.kg^−1^.min^−1^. One later study on American bison reports that the use of 2 L.min^−1^ (no weight reported) allows to improve P_a_O_2_ but not to resolve hypoxemia in two bison (8). Another one reported the use of 8–10 L.min^−1^ for 21 individuals ranging from 300 to 800 kg but does not evaluate the benefits on blood oxygenation (16). Using a higher nasal insufflation rate would allow a better flush of the anatomical dead space, increasing the available oxygen concentration and volume for inhalation, and would better match the inspiratory flow of bison, leading to an increased fraction of inspired oxygen, although higher flow rates can exacerbate hypercapnia (65–67). The respiratory rate, the breathing pattern, and the delivery method also contribute to the efficiency of oxygen supplementation (47, 67, 68). Yet, we strongly recommend investigating higher delivery rates while balancing hypercapnia for relieving hypoxemia in immobilized European bison.

We observed a significant increase in P_a_CO_2_ during oxygen supplementation. This is partly attributed to the hypoventilation, and the fact that we did not use mechanical ventilation to compensate for the drug-induced depression of receptors sensitive to P_a_CO_2_, the hypercapnia continued to increase with time in immobilized animals (47). Additionally, oxygen delivery can increase the degree of hypoventilation in immobilized ruminants (38, 69). This would be explained by the combination of the Haldane effect, resulting in decreased affinity of hemoglobin for CO_2_ in oxygenated blood, and an impairment of hypoxic pulmonary vasoconstriction (70). However, for ethical reasons, we did not include a control group (i.e., without oxygen) and cannot assess the actual effect of the oxygen supplementation on hypoventilation. The degree of mild hypercapnia observed in our study is reported to be generally well-tolerated in ruminants (41). This permissive hypercapnia can potentially have some beneficial effects during the immobilization by supporting cardiovascular function. This is possible through a positive inotropic effect, improving ventilation-perfusion matching and brain perfusion, and by favoring the release of oxygen from hemoglobin to the tissue (71). Yet severe or prolonged hypercapnia might result in deleterious effects on cardiovascular and neurological function including increased intracranial pressure, impaired myocardial contractility, narcosis, and coma (72). Furthermore, hypercapnia is also responsible for the development of respiratory acidosis (73). In our study, the mild hypercapnia observed led to lowered mean pH value and subsequent respiratory acidosis in several bison. The pH levels observed here are similar to those reported for American bison immobilizations (8, 57) as well as in other ungulate species (37, 38, 40). The increased mean level of bicarbonate observed probably resulted from a metabolic compensation to respiratory acidosis (73). In our study, acidemia and hypercapnia remained within a mild range. Nevertheless, severe acidemia can result in serious detrimental effects on cardiovascular function affecting myocardial contractility, arterial blood pressure, and predisposing to arrhythmia and ventricular fibrillation (74). This must be considered when increasing oxygen flow rates, and careful titration with blood gas analyses are recommended. The low lactate values, together with the absence of observed hyperthermia (75), are likely reflective of calm inductions (7, 76, 77).

We observed increased recovery time for individuals with lower rectal temperatures. This can possibly be explained by a reduction of drug metabolism in animals with low body temperature (75, 78). However, this result should be considered with care due to our small sample size and the relatively narrow range of temperature recorded. Furthermore, according to model selection, both the total dose of etorphine and the immobilization time might have influenced our results, although not having a significative effect in the rejected model. Further study, especially design to test this hypothesis, would be needed to assess the factors influencing recovery. Overall, we recorded rectal temperatures close to the average rectal temperature reported for European bison [38.5 ± 2°C (48); 38.7°C (49)] as well as relatively slow recovery time (11.4 ± 7.5 min) using diprenorphine as antidote to etorphine. This is close to the recovery time reported for xylazine-zolazepam-tiletamine combination [11.8 ± 9.7 min (6)], but slower than butorphanol-azaperone-medetomidine [4.9 ± 2.8 min (16)], nalbuphine-medetomidine-azaperone [4.0 ± 1.1 min (8)], carfentanil-xylazine [4.1 ± 1.6 min (54)], and medetomidine-zolazepam-tiletamine [1.7 ± 0.8 min (6)] combinations. The somewhat slow recovery time observed in our study can be explained by the use of diprenorphine as an antidote, a partial antagonist of etorphine as compared to naltrexone, which is a full antagonist (13). Earlier uses of naltrexone for reversal at Avesta sometimes led to sudden and rough recoveries, with stressed bison, running into fences and posing a risk for animals and handlers safety (S. Björck, unpublished data, 2022). The recovery time being still quite short with diprenorphine but with calmer recovery, we would therefore recommend this antidote in a captive setting, while naltrexone could be more useful for free-ranging individuals. We also found some important variations in recovery time between individuals (2–38 min). Although recovery remains fast and suitable in a captive setting, this variation can be an issue when planning for field immobilization. We did not identify a relationship between hypoxemia and the recovery time, as earlier reported in reindeer immobilized with the same drug combination (38). This is possibly because we only measured P_a_O_2_ twice during the immobilization event, not allowing an accurate averaging of P_a_O_2_ throughout the immobilization. Differences observed can also come from the use of different antidotes (naltrexone hydrochloride and tolazoline hydrochloride).

Regurgitation during immobilization is a well-known side effect of both opioids and alpha-2 adrenoceptor agonists through cardiac sphincter relaxation and unsafe positioning (79). Despite the care given to bison’s sternal position, three out of the 39 immobilized animals presented with minor regurgitation, leading to a small amount of ruminal fluid in the mouth and the nostrils. The presence of ruminal content in the mouth and nostrils is considered a concern during immobilization as it can lead to aspiration and consequent pneumonia (15). To reduce this risk, it is recommended, when possible, to fast the animals before the immobilization, and to position the animal in sternal recumbency with the head held high and the mouth and nose pointing downward (24). For animals with a risk of aspiration, long-acting antibiotics with a broad spectrum were administered prophylactically (79) and no signs of pneumonia were reported for at least 2 months.

Between 2008 and 2022, 189 immobilizations of European bison were carried out at Avesta Visentpark, including 60 for loading the animals onto transport vehicles, following basically the same protocol that we described (without acepromazine in some cases and some slight dose variations initially). The absence of mortality or obvious complications post immobilizations in our study and during all those events (S. Björck, unpulblished data, 2022) are an encouraging output and reflect a sufficient degree of safety associated with this protocol. However, immobilization remains a stressful event with a cost for the animals that occurs in addition to other sources of stress, which may affect the animal’s general well-being and potential translocation success (80, 81). Additional studies documenting the efficiency and side effects of this protocol will greatly benefit the overall knowledge and safety regarding European bison immobilization.

## Conclusion

Our results indicate that the combination of etorphine-acepromazine and xylazine provides a sufficient level of immobilization for captive European bison for diverse common management and husbandry procedures. However, the immobilization process is associated with the development of mild to severe hypoxemia, a small risk of regurgitation and mild respiratory acidosis. This acidosis is most likely driven by hypoventilation and ventilation-perfusion mismatch, and potentially accentuated by oxygen supplementation. Our recommended dose is 0.015 mg.kg^−1^ etorphine, 0.049 mg.kg^−1^ acepromazine, and 0.22 mg.kg^−1^ xylazine since this dose induced recumbency and sedation using one dart.

When using this protocol, we recommend fasting the animals the morning of the immobilization or the evening before and positioning them in sternal recumbency for the procedure. We strongly recommend implementing oxygen supplementation with more than 1 L.min^−1^.100 kg^−1^ and a close monitoring of respiratory parameters. Further studies are required to properly assess a more accurate flow rate for resolution of hypoxemia in immobilized bison. Likewise, considering the study setting, our recommendations apply for captive European bison. Further studies are warranted to determine appropriate doses for free-ranging animals.

## Data availability statement

The raw data supporting the conclusions of this article will be made available by the authors, without undue reservation.

## Ethics statement

Ethical review and approval was not required for the animal study because all samples used in this study were collected as part of a standardized routine animal anesthesia monitoring protocol during chemical immobilization of bison, either for management, veterinary care or for translocation purposes (as per compulsory EU health regulations, approved by Avesta Visentpark).

## Author contributions

NG participated to the data collection, data analysis and interpretation, preparation of the first manuscript, and revision manuscript. ML and SB participated to conceptualize the study design, to data collection, helped to prepare the first manuscript, and to the revision of the manuscript. JA and AE participated to conceptualize the study design, to data collection, and to the revision of the manuscript. JM, DB, RW, AG, and AT participated to the data collection and the revision of the manuscript. All authors contributed to the article and approved the submitted version.

## Funding

This work was funded by internal funding from the One Health and Ecophysiology Research Group, and from the master thesis funding grant supported by Department of Forestry and Wildlife Management, Faculty of Applied Ecology, Agricultural Sciences and Biotechnology, Inland Norway University of Applied Sciences, Campus Evenstad, Koppang, Norway.

## Conflict of interest

The authors declare that the research was conducted in the absence of any commercial or financial relationships that could be construed as a potential conflict of interest.

## Publisher’s note

All claims expressed in this article are solely those of the authors and do not necessarily represent those of their affiliated organizations, or those of the publisher, the editors and the reviewers. Any product that may be evaluated in this article, or claim that may be made by its manufacturer, is not guaranteed or endorsed by the publisher.
