# Supplementary material for: Arterial oxygenation and acid–base status before and during oxygen supplementation in captive European bison (Bison bonasus) immobilized with etorphine-acepromazine-xylazine
Source: Front Vet Sci. 2023 Jun 12;10:1125919. doi: 10.3389/fvets.2023.1125919 (PMC10296773; doi:10.3389/fvets.2023.1125919)
Supplement: Supplementary file 1 [file Table_1.DOCX]

| Parameter  (unit) | Model family (link) | Sample size | variable  (unit) | Estimate | 95% CI | p-value | r^2^ |
| --- | --- | --- | --- | --- | --- | --- | --- |
| Recovery time  (min) | Gamma  (log) | 33 | Intercept | 20.96 | [8.20, 33.86] | 0.005 | 0.13 |
|  |  |  | Temperature  (°C) | -0.48 | [-0.81, -0.15] | 0.011 |  |
| Etorphine start dose  (mg/kg) | Gaussian (identity) | 26 | Intercept | 0.015 | [0.014, 0.017] | < 0.001 | 0.52 |
|  |  |  | Several injections’ group | -0.006 | [-0.008, -0.003] | < 0.001 |  |
| Xylazine  start dose  (mg/kg) | Gaussian (identity) | 26 | Intercept | 0.22 | [0.20, 0.24] | <0.001 | 0.20 |
|  |  |  | Several injections’ group | -0.04 | [-0.07, -0.01] | 0.022 |  |

Additional file 1. Results of the regression analyses for the best models

The variable called “several injections’ group” corresponds to a categorical variable with two levels: one is the group of bison that have required more than one injection during the whole procedure and the other one is for those that got only one injection.
