# Supplementary material for: Arterial oxygenation and acid–base status before and during oxygen supplementation in captive European bison (Bison bonasus) immobilized with etorphine-acepromazine-xylazine
Source: Front Vet Sci. 2023 Jun 12;10:1125919. doi: 10.3389/fvets.2023.1125919 (PMC10296773; doi:10.3389/fvets.2023.1125919)
Supplement: Supplementary file 2 [file Table_2.docx]

| *Variable* | *Unit* | *n_t1_* | *Mean ± SD*  *(min – max)* | *Trend* | *n_t2_* | *Mean ± SD*  *(min – max)* | *p-value*  *(n)* |
| --- | --- | --- | --- | --- | --- | --- | --- |
| Pulse rate | Beats.min^-1^ | 39 | 54 ± 8  (32-76) | = | 29 | 52 ± 9  (32-72) | 0.313^1^  (n = 28) |
| Respiratory rate | Breath.min^-1^ | 39 | 11 ± 8  (2 - 36) | = | 29 | 9 ± 4  (4 - 16) | 0.177^2^  (n = 28) |
| Rectal  tem­perature | °C | 39 | 38.7 ± 0.6  (37.3 – 39.7) | = | 32 | 38.7 ± 0.6  (37.5 – 39.8) | 0.442^2^  (n = 31) |
| pH ^*a^ |  | 35 | 7.36 ± 0.05  (7.22 – 7.48) |  | 32 | 7.33 ± 0.05  (7.22 – 7.46) | < 0.001^1^  (n = 31) |
| P_a_O_2_ ^*a^ | mmHg | 35 | 49.7 ± 18.3  (21.0 – 101.0) |  | 32 | 90.2 ± 28.3  (49 - 165) | < 0.001^1^  (n = 31) |
| P_a_CO_2_ ^*a^ | mmHg | 35 | 52.2 ± 7.5  (36.1 – 64.3) |  | 32 | 59.0 ± 8.2  (36.3 - 71.8) | < 0.001^1^  (n = 31) |
| HCO_3_^- a^ | mmol.L^-1^ | 35 | 28.8 ± 3.1  (22.3 – 34.5) |  | 32 | 30.1 ± 3.4  (21.4 – 36.0) | 0.003^2^  (n = 31) |
| BE | mmol.L^-1^ | 35 | 7.2 ± 10.8  (-3.0 – 36.0) | = | 32 | 4.0 ± 3.4  (-5.0 – 10.0) | 0.653^2^  (n = 31) |
| Lactate ^a^ | mmol.L^-1^ | 34 | 1.18 ± 0.90  (0.30 – 4.02) |  | 32 | 0.85 ± 0.54  (0.30 – 2.42) | 0.001^2^  (n = 30) |

*^*^: temperature corrected values; ^a^: significant difference after oxygen supplementation. t_1_ and t_2_ correspond to the time from when the animal is down to respectively the 1^st^ and the 2^nd^ sample. ∆_t_ is the time between the first and the second sample. For every parameter, the mean ± SD (min-max) and the number of animals (n_t_) are reported. The p-values are the results of paired t-tests between t_1_ and t_2_ for each parameter on n individuals. ^1^: two-tail paired t-test; ^2^: Wilcoxon signed-rank test.*

*P_a_O_2_: partial pressure of oxygen in arterial blood ; P_a_CO_2_: partial pressure of carbon dioxide in arterial blood ; HCO_3_^-^: concentration of bicarbonate in arterial blood ; BE: Base excess*

Table 2. Physiological parameters from captive European bison (Bison bonasus) during chemical immobiliza­tion using etorphine-acepromazine-xylazine.
